# Isolation and Characterization of a Lytic Phage PaTJ Against *Pseudomonas aeruginosa*

**DOI:** 10.3390/v16121816

**Published:** 2024-11-21

**Authors:** Jiayu Gu, Xinqiao Zhang, Tianlang Liu, Yunxue Guo

**Affiliations:** 1 Key Laboratory of Tropical Marine Bio-Resources and Ecology, South China Sea Institute of Oceanology, Chinese Academy of Sciences, Guangzhou 510000, China; gujiayu20@mails.ucas.ac.cn (J.G.); liutianlang18@mails.ucas.edu.cn (T.L.); 2 University of Chinese Academy of Sciences, Beijing 100000, China; 3 Frontiers Science Center for Synthetic Biology (Ministry of Education), Tianjin University, Tianjin 300000, China; 2022207652@tju.edu.cn

**Keywords:** *Pseudomonas aeruginosa*, lytic phage, metabolism, type IV pili

## Abstract

*Pseudomonas aeruginosa* is a major global threat to human health, and phage therapy has emerged as a promising strategy for treating infections caused by multidrug-resistant pathogens. In this study, we isolated and characterized a *Pseudomonas* lytic phage, PaTJ, from wastewater. PaTJ belongs to the phage family *Mesyanzhinovviridae*, and is featured by short latency (30 min) and large burst size (10^3^ PFU per infected cell). Our investigation revealed that PaTJ utilizes the type IV Pili (T4P) as a receptor. Transcriptome analysis of PaTJ infected host at latent stage showed distinct expression patterns of PaTJ encoding genes involved in replication and structure assembly, without expression of the majority of toxic accessory genes responsible for phage release. In addition, host bacteria exhibited specific induction of host metabolism-related genes in response to the PaTJ’s infection. Furthermore, our findings demonstrated the PaTJ’s potential in degrading biofilms. This work sheds light on the multifaceted impact of this lytic phage PaTJ on *P. aeruginosa*, presenting potential applications in both gene expression modulation and biofilm management.

## 1. Introduction

Antibiotics have been globally utilized and have significantly contributed to the advancement of healthcare clinics, veterinary practices, agriculture, and the food processing industries since the discovery of penicillin in 1928 [1]. However, the widespread and indiscriminate use of antibiotics over time has led to the emergence of multidrug-resistant bacteria [2]. These bacteria have developed various mechanisms to evade the effects of antibiotics, including the utilization of ‘intrinsic’ genes they already possessed or the acquisition of ‘acquired’ resistance genes through horizontal gene transfer and the formation of resilient biofilms [3,4]. Consequently, antimicrobial resistance has disrupted the natural microbial balance in various ecosystems, potentially leading to long-term ecological consequences. Compounding this issue is significant decline in the sustainable discovery and development of new antibiotics, exacerbating the challenge of combating resistant pathogens. In recent decades, there has been an urgent need worldwide to address the public health menace posed by antimicrobial resistance [5], and to develop alternative strategies to effectively treat bacterial infections while mitigating the spread of antibiotic resistance. These alternative strategies include phage therapy, CRISPR-Cas9 technology, improving the use of current drugs, and the exploration of natural compounds [3].

Phages are viruses that can infect and finally kill host bacteria. Phage therapy, which uses phages to treat bacterial infections, has been around for over a hundred years [6]. In the past 20 years, support from researchers and doctors has pushed the development of this field as a promising therapy in the context of the rise of antimicrobial resistance worldwide [7]. Phage cocktails combined with antibiotics have been applied in the treatment of multiple antibiotic resistance strains due to escape colonies usually appearing and becoming dominant, and successful cases of phage therapy have been reported in different regions [8,9,10,11,12,13]. However, several factors including the efficiency of killing, whether the phages are lytic or not, and the side effects of phages, should be taken into consideration before the use of phage for treatment [14]. Thus, more phage resources should be isolated and their infection model should be elucidated before using it in phage therapy.

*Pseudomonas aeruginosa*, a versatile and ubiquitous Gram-negative bacterium, holds significant importance in diverse environments, including soil, water, and even medical equipment surfaces [15]. This pathogen poses a serious threat because it has developed resistance mechanisms through various genetic adaptations, including the production of enzymes that degrade antibiotics, efflux pumps that expel drugs from the cell, and alterations in drug targets to reduce antibiotic efficacy [16,17]. Moreover, *P. aeruginosa*’s ability to form biofilms further enhances its resistance by providing a protective environment for bacterial colonies to thrive and resist antibiotic penetration [18,19]. The emergence of multidrug-resistant strains underscores the urgency in developing novel therapeutic strategies to combat infections caused by this resilient pathogen. A series of phages infecting *P. aeruginosa* have been isolated and some have been used in cocktail preparation [8,20,21,22,23,24,25].

This study focused on the isolation and characterization of a *Pseudomonas* phage, PaTJ (*P. aeruginosa* phage from Tianjin), which utilizes type IV pili (T4P) as its receptor. Belonging to the *Mesyanzhinovviridae* family, this phage encodes a variety of structural and accessory genes. These genes exhibit differential expression patterns during phage infection and propagation, significantly promoting the metabolism-related pathways of the host strain. Moreover, the phage demonstrates efficient degradation of *Pseudomonas* biofilms, suggesting its potential as a valuable component in phage cocktails designed to target *Pseudomonas* spp. containing intact T4P.

## 2. Materials and Methods

### 2.1. Bacterial Strains and Culture Conditions

The strains, plasmids, and primers used in this study are listed in Table S1*. P. aeruginosa* strain MPAO1 [26] was used as a host strain for phage isolation and propagation. MPAO1 Δ*pilC* and Δ*pilA* were used for the determination of receptor PaTJ. All the strains were cultured in LB medium [27] at 37 °C at 200 rpm except the *E. coli* conjugation strain WM3064 with 300 µM diaminopimelic acid (DAP) in LB medium.

### 2.2. Phage Isolation and Purification

Phage was isolated from wastewater collected from Institute of Hematology and Blood Diseases Hospital, Tianjin, China. Briefly, samples were filtered through a 0.22 μm microporous membrane to remove bacterial cells. Overnight, *P. aerugionosa* MPAO1 was adjusted to OD_600_~0.1 and then 100 μL filtered sewage was added to 1 mL MPAO1 host bacteria to co-culture for 6–8 h at 37 °C. The mixture was diluted carefully and each dilution was mixed with 4 mL R-top medium (1 g Tryptone, 0.1 g Yeast, 1 g NaCl per 100 mL) followed by pouring into the LB agar. The double-layer plate was stored at 37 °C for 8 h. Plaque was screened and stored in SM buffer (10 mM Tris-HCl, pH 7.5; 100 mM NaCl; 10 mM MgSO_4_) at 4 °C.

### 2.3. Transmission Electron Microscopy (TEM)

About 3 µL of purified bacteriophage suspension was gently placed on glow-discharged carbon-coated 300 mesh copper grids. After about 1 min, the remaining solution on the grids was wicked away with the help of a filter paper. The grid was then stained with 2% (wt/vol) uranyl acetate and air-dried. The negatively stained phage particles were visualized with a FEI Tecnai 12 BioTwin Transmission electron microscope (FEI, Ermelo, The Netherlands) at an operating voltage of 100 kV. Virus particle dimensions were measured using the ImageJ computer program, with the software scale set on the scale bar obtained from the electron micrographs.

### 2.4. One-Step Growth Curve

The one-step phage growth was performed as previously described [28]. In brief, 10 mL *P. aeruginosa* culture (OD = 1.0) was infected with phage PaTJ at a multiplicity of infection (MOI) of 0.1 and adsorbed for 15 min at 37 °C. Then, cells were centrifuged in 12,000× *g* for 5 min, and unadsorbed phages were removed carefully and washed with fresh LB medium. Cell pellets were then resuspended in 10 mL fresh LB broth and incubated at 37 °C at 200 rpm. Cultures were collected for double-layer plaque assay at 0, 10, 20, 30, 40, 50, 60, 90, and 120 min.

### 2.5. Gene Knockout in P. aeruginosa MPAO1

The *pilC* and *pilA* genes were deleted following previously established protocols [29]. All primers utilized in this procedure are detailed in Table S1. In detail, the upstream and downstream fragments of *pilC* and *pilA* were PCR-amplified from MPAO1 genomic DNA and subsequently inserted into the modified suicide plasmid pEX18Gm digested with EcoRI and HindIII. These constructs were then introduced into WM3064 and transferred via conjugation into MPAO1 strains. In-frame deletion mutants were obtained using the sucrose resistance selection method. The accuracy of the final deletion mutants was confirmed by PCR with gene-specific primers (gene-SF/SR, gene-LF/LR) and DNA sequencing.

### 2.6. Killing Dynamics

Overnight, wild-type MPAO1 culture was diluted 1000 folds, and mixed with PaTJ phages at various MOIs (1000, 100, 10, 1, 0.1, and 0.00001). Then, the mixtures were seeded in 96-well plates with 200 μL per well. The plates were cultured at 37 °C and cell turbidity was measured each minute till 16 h. The growth of MPAO1 cells without adding phages (MOI = 0) was used as a control.

### 2.7. Phage Genome Isolation, Sequencing, Annotation, and Comparative Genome Analysis

PaTJ phage genomic DNA was isolated using the TIANamp Virus DNA Kit (Tiangen, Nanjing, China) according to the manufacturer’s procedure. Libraries were prepared for Illumina paired-end sequencing (PE150), and sequenced by Shanghai Biozeron Biotechnology Co., Ltd. (Shanghai, China). The genome was annotated with the RAST annotation engine (https://rast.nmpdr.org/, accessed on 20 October 2023) [30]. Comparative genome analysis among four phages was conducted using Easyfig_2.2.5_win software.

### 2.8. Genome Comparison

Firstly, the genomes of the three most closely related homologous phages of PaTJ phages were collected, and the orientations of these four phage genomes were corrected and aligned, respectively, so that their orientations were all forward and the starts were all DNA helicase. Then, based on the annotation results, the annotation results were classified into six categories, and the genome comparison map was drawn using the R package gggenome.

### 2.9. Phylogenetic Tree Analysis

Firstly, 22 homologous phage genomes of PaTJ phages were retrieved from the NCBI Virus database (https://www.ncbi.nlm.nih.gov/labs/virus/vssi/#/, accessed on 15 August 2024). In addition, 2 outgroups including *P. aeruginosa* phage phi3 (P2 familay) and *P. aeruginosa* phage Pa-O (PAcq-4) were collected. Whole genome sequences were subjected to MUSCLE alignment using MEGA11, and the tree was constructed using the neighbor-joining method.

### 2.10. RNA Isolation and Strand-Specific RNA Sequencing

Overnight, MPAO1 cultures were diluted to OD_600_ 0.1 and incubated to OD_600_ 1.0. Then phage PaTJ was added at MOI 0.01, and the obtained phage–bacteria mixtures were cultured for 30 min before sample collections. A volume of 1 mL liquid cultures was collected by centrifugation with 12,000× *g* at 4 °C. The total RNA in cell pellets was isolated using the Bacteria RNAprep Pure Kit (Tiangen Biotech Co. Ltd., Beijing, China) according to the manufacturer’s instructions. The quality of total RNA was determined with Agilent RNA ScreenTape (Lot: 0201937-152). The RNA with high-quality experienced rRNA was removed with the Ribo-off rRNA Depletion Kit (Bacteria) (Vazyme, # N407-C1). An amount of 1 µg total RNA was used for cDNA library preparation. There were 11 amplification cycles, and the following steps were performed using the VAHTS Total RNA-seq (Bacteria) Library Prep Kit for Illumina, San Diego, CA, USA, (Lot:7E582A1) according to the manufacturer’s instructions. Subsequently, the libraries were sequenced on a NovaSeq 6000 S4 (Illumina) and the raw data were evaluated with FastQC (v0.10.1). After removing the contamination and adapter sequences with Cutadapt (version 1.9.1), the obtained clean data were mapped to the phage PaTJ genome and MPAO1 genome (GenBank accession number: CP079712), separately. Then, the expression profiling was calculated with Htseq (V 0.6.1). The differential gene expression (DGE) analysis was performed using EdgeR [31] (R-3.1.2), and significantly changed genes (≥2-fold and FDR ≤ 0.05) in the PaTJ infection group compared to the PaTJ non-infection group were selected for further analysis.

### 2.11. Biofilm Degradation

The experiment was conducted as we described previously [26]. In brief, a volume of 200 μL aliquots of MPAO1 (OD_600_~0.1) was incubated in 96-well microtiter plates for 24 h to form statically growing biofilms. Then, the planktonic cells were removed carefully and 200 μL of PaTJ diluted in LB broth was added to each well at MOI 0.1. The biofilm was determined at 0, 4, and 8 h post phage infection by crystal violet staining.

## 3. Results

### 3.1. Plaque and Phage Morphology of PaTJ

**Figure 1:**
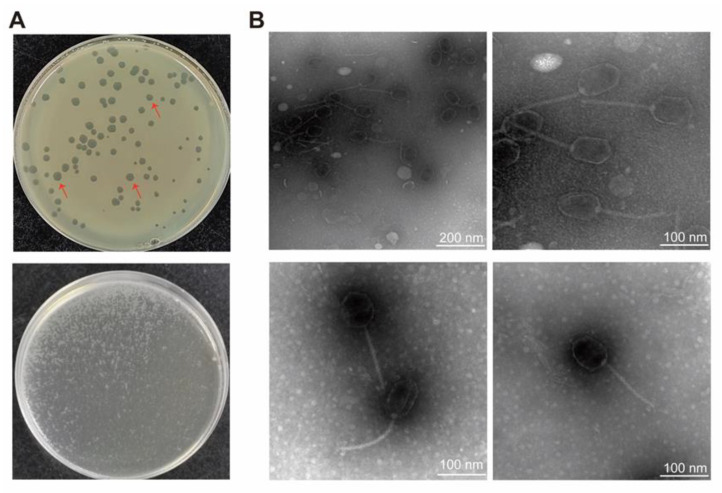
Identification and morphology of *Pseudomonas* phage PaTJ. (**A**) Plaques for the isolation of *Pseudomonas* phage PaTJ on a wild-type MPAO1 lawn after double layer agar plating (top), and the PaTJ plaques selected for further phage propagation were indicated by red arrows. The plate used for the preparation of high titer phage lysate in this study (bottom). (**B**) TEM images of negatively stained phage PaTJ particles are presented at two magnifications. Representative images were shown.

Based on the double-layer plate method, *P. aeruginosa* phages were isolated by screening against the model strain MPAO1 using wastewater collected at the Institute of Hematology and Blood Diseases Hospital, Tianjin, China. Among them, a phage PaTJ with the largest ‘bull eye morphology’ was selected. Following repeated purification and incubation at 37 °C for 8 h, the majority of PaTJ plaques showed stable regular dots of 3–5 mm in diameter (Figure 1A). Subsequently, three large and clear plaques were selected for further enrichment to obtain a high-titer lysate (~10^10^ PFU mL^−1^) (Figure 1A).

TEM analysis of the purified phage particles, following the guidelines of the International Committee on Taxonomy of Viruses (ICTV), revealed that PaTJ belongs to the bacteriophage family Mesyanzhinovviridae. This classification is supported by the presence of a head, neck, contractile tail, base plate, and tail fiber geometry (Figure 1B). The average (n = 10) particle length (head to be base plate), head diameter, head length, tail length, and tail diameter were measured to be 225.58 ± 9.87, 53.95 ± 3.66, 82.06 ± 6.35, 141.05 ± 3.35, and 7.91 ± 0.72 nm, respectively.

### 3.2. One-Step Growth Curve and Killing Kinetics of PaTJ

**Figure 2:**
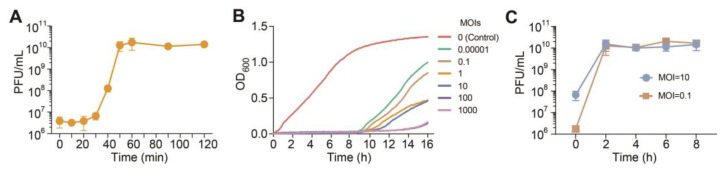
One-step growth curve and killing kinetics of PaTJ. (**A**) The one-step growth curve of PaTJ on the *P. aeruginosa* strain MPAO1 was conducted at MOI 0.1, and three independent cultures were used. The data are presented as mean ± SD. (**B**) Killing curves of *P. aeruginosa* strain MPAO1 by PaTJ were generated at various MOIs (1000, 100, 10, 1, 0.1, and 0.00001). The growth of MPAO1 cells without the addition of phages (MOI = 0) was used as the control. (**C**) Phage titers in cultures in B at MOIs 10 and 0.1, and the data are presented as mean ± SD.

Evaluation of latent period and burst size by determining the one-step growth is usually conducted for newly isolated phages. Here, we observed that the multiplicity of infection (MOI) when the highest titer of phage lysate was obtained is 0.1, and we therefore performed the one-step growth curve at MOI = 0.1 for 10 min before washing unabsorbed PaTJ phages. As a result, the latency period of PaTJ is 30 min (Figure 2A). Afterward, the phage titers in the cultures increased logistically till 50 min when the number of phages in the culture began to stabilize, reaching 10^10^ PFU mL^−1^. In addition, the burst is about 10^3^ average progeny per infected cell (Figure 2A).

To evaluate the lytic activity of PaTJ against *P. aeruginosa*, MPAO1 bacterial growth was assessed in a 96-well plate at different MOIs (Figure 2B). The results indicated a typical sigmoid curve representing uninhibited bacterial growth, while at specific MOIs, bacterial growth was completely inhibited, with as low as MOI = 0.00001 showing no growth of cells for up to 8 h (Figure 2B), indicating the strong lysis ability of PaTJ. Furthermore, the PFU of PaTJ at MOI 10 and 0.1 initially showed logistic growth in the first 2 h and then remained relatively stable (Figure 2C). However, as the infection progressed, bacterial cells began to grow, albeit at a significantly slower rate than the non-phage infection control, suggesting the emergence of resistant bacterial strains. This underscores the need for caution in prolonged treatment with lytic phages and indicates that higher MOIs do not necessarily yield better outcomes.

### 3.3. Receptor of Phage PaTJ

**Figure 3:**
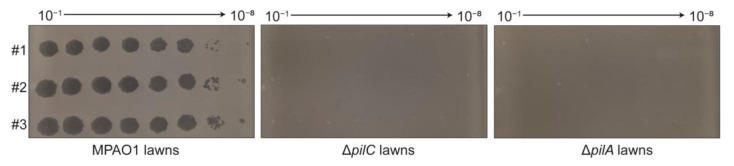
PaTJ uses T4P as its receptor. The PaTJ phages were serially diluted by a factor of 10 and were plated onto lawns of the wild-type MPAO1 strain as well as two T4P mutant strains, Δ*pilC* and Δ*pilA*, respectively. Three independent phage samples were utilized and were designated as #1, #2, and #3.

Many phages, including *P. aeruginosa* lytic tailed phages QDWS [32], D3112 [33], phiKMV, and MPK7 [34], as well as ssDNA filamentous phages Pf4 [35] and RNA phage PP7 [36], all require T4P for infection. Here, we infected the wild-type MPAO1 as well as two T4P mutant strains, Δ*pilC* and Δ*pilA*, in which the genes encoding the T4P platform protein and filaments were deleted separately (Figure 3). The results demonstrated that PaTJ was unable to infect the two mutants at all, while efficiently infecting the MPAO1 wild-type strain. This finding indicates that PaTJ utilizes T4P as its receptor for the initiation of adsorption and subsequent infection.

### 3.4. Genomic Features of Phage PaTJ

PaTJ is a double-stranded DNA phage with a total genome size of 61,922 bp and a GC content of 64.53%. CARD (Comprehensive Antibiotic Resistance Database) annotation and VFDB (Virulence Factor Database) comparison showed that no antibiotic resistance genes or virulence genes were encoded by PaTJ, suggesting that this phage is safe.

The RAST (Rapid Annotation using Subsystem Technology) annotation, combined with Uniprot BLAST, identified 90 functional ORFs (Supplementary Table S2). Among these, 35 are diverse structural phage proteins, including the major capsid protein (PaTJ_15), minor head protein (PaTJ_17), and five tail fiber-related proteins. Notably, PaTJ_51 and PaTJ_52 are integrase and Lambda repressor-like proteins, respectively, with the latter containing a DNA-binding domain (6–93 aa), both of which are critical for prophage maintenance and integration into the host chromosome as a prophage. This suggests that PaTJ may have the potential ability to integrate into the bacterial chromosome. Additionally, PaTJ_56 functions as a DNA primase, catalyzing the synthesis of short RNA molecules used as primers for DNA polymerases, and its continual activity is required at the DNA replication fork. PaTJ_57 is a DNA polymerase and PaTJ_34 is an RNA polymerase-binding protein, potentially responsible for PaTJ genome DNA replication and the transcription of PaTJ encoding genes, respectively. Furthermore, no tRNAs were identified in the genome of PaTJ, indicating that this phage undergoes DNA replication with its own replication system and further expression by hijacking the host’s expressing machine. PaTJ_75 (397 aa in length) is a UvrD-like helicase belonging to helicase superfamily I, while PaTJ_79 (564 aa in length) is a helicase C-terminal domain-containing protein belonging to superfamily II helicase. Despite no identified similarity between the two helicases, they shared a catalytic core (such as GTGK and DE motifs) with high structural similarity. These two helicase proteins may coordinately resolve common issues that arise during PaTJ phage DNA replication, recombination, and repair. Additionally, PaTJ_20 and PaTJ_21 encode the phage terminase large subunit and small unit, respectively. A function of the small terminase is to initiate the packaging of the viral genome, while the large terminase is responsible for the ATP-powered translocation of DNA. Furthermore, PaTJ phage encodes the HicA family toxin of type II toxin-antitoxin system *hicAB* (no *hicB* homolog was identified by both annotation and further analysis of predicted ORFs upstream and downstream), along with some other potential toxic proteins such as endonuclease (PaTJ_30, PaTJ_35, PaTJ_67), lysin (PaTJ_9), and holin (PaTJ_10). These proteins may function in lysing the host cell during the release process by forming micron-sized pores in the bacterial inner membrane and releasing endolysin into the periplasmic space to degrade peptidoglycans. Moreover, several other accessory genes and 21 hypothetical proteins were also identified, which may confer new characteristics to PaTJ in its life cycle and contribute to phage-host interactions.

**Figure 4:**
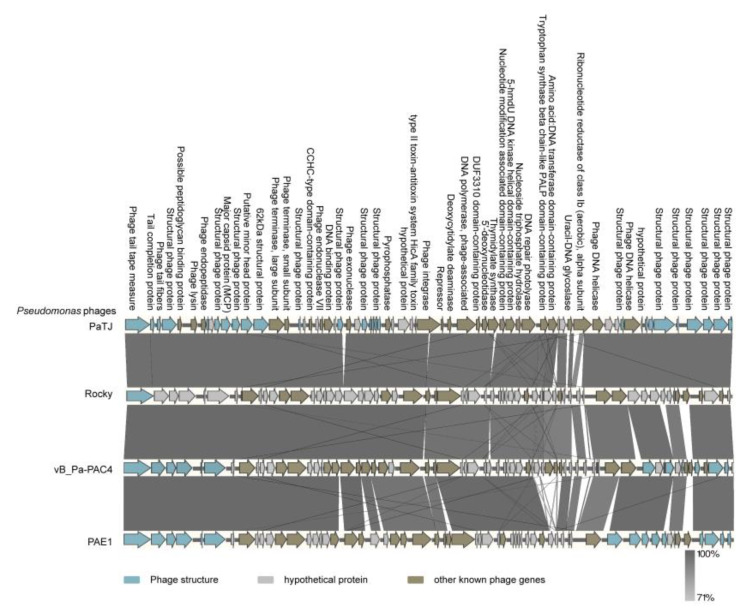
Comparative genome analysis of the *P. aeruginosa* phages PaTJ, Rocky, vB-Pa-PAC4, and PAE1.

**Figure 5:**
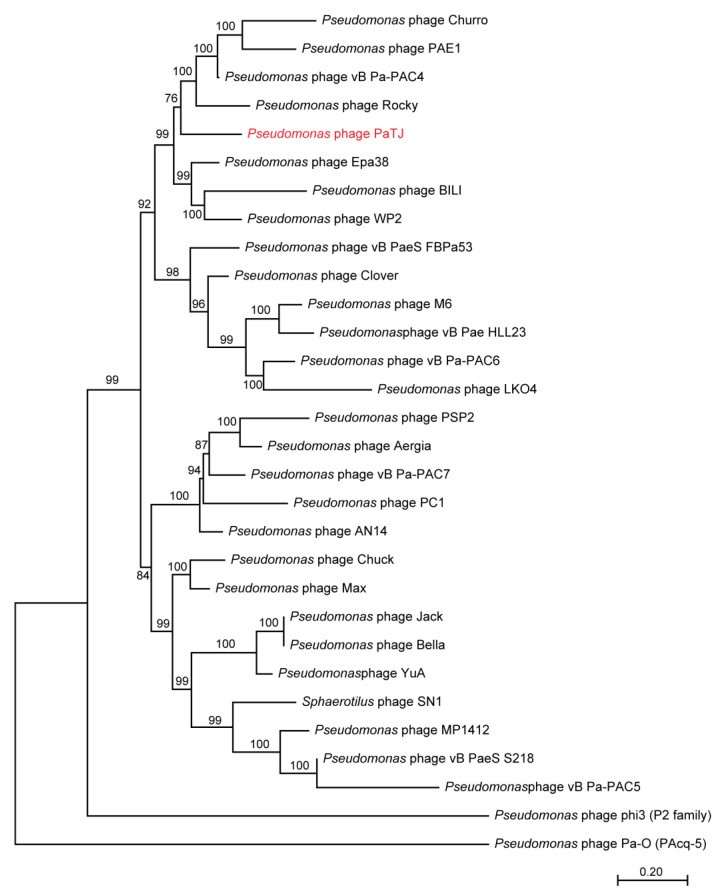
The maximum likelihood tree based on the whole genome sequences of phage PaTJ (red) and the available phages listed in Supplementary Table S2.

Phage PaTJ was relatively similar to *Pseudomonas* phage Rocky (97%, coverage, 97% identity, accession number PP661415.1), vB Pa-PAC4 (95%, coverage, 98% identity, accession number PP408251.1), and PAE1 (93%, coverage, 98% identity, accession number PP408251.1), indicating that these phages may belong to the same genus. Easyfig analysis further showed that PaTJ was highly homologous to the aforementioned phages (Figure 4). Phylogenetic analysis of the whole genome with 29 other *Pseudomonas* phages also showed that PaTJ fell in the clade with the family *Mesyanzhinovviridae* (Figure 5, Supplementary Table S3).

### 3.5. Expression Pattern of PaTJ Encoding Genes During Infection

**Figure 6:**
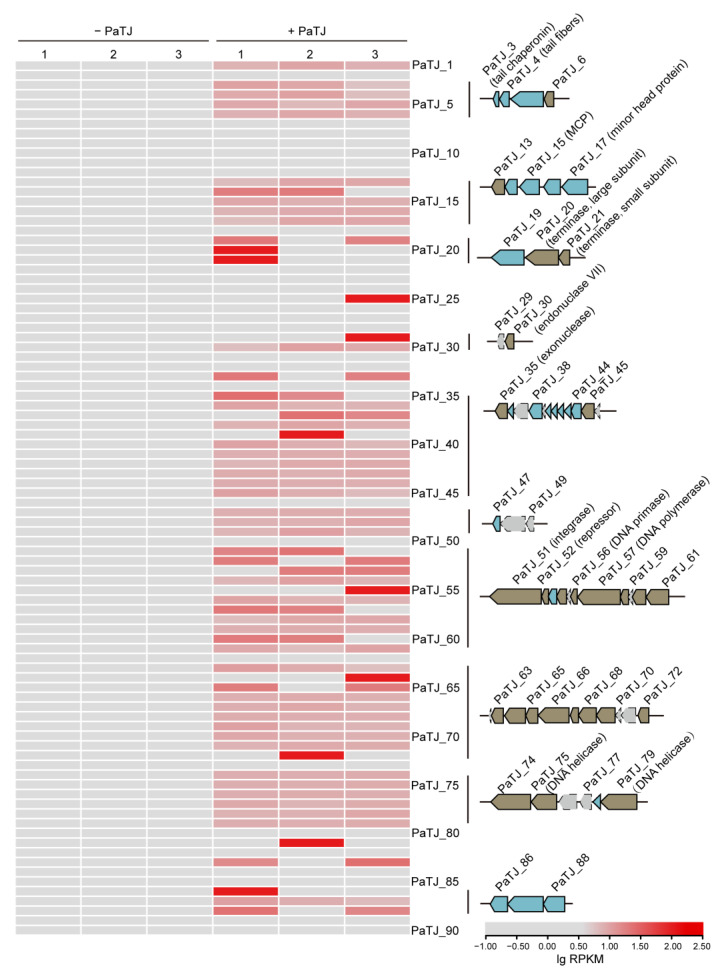
MPAO1 cells were infected with phage PaTJ at an OD_600_ of 1.0 with MOI 0.01 for 30 min. Normalized gene expression (lgRPKM) of all PaTJ-encoding genes was shown on the left. The gene clusters with continuous similar expression patterns were also depicted on the right, and genes encoding hypothetical proteins were shown in gray. Phage structural proteins were depicted in blue, while those encoding homologs of known phage accessory proteins were depicted in brown. MCP, major capsid protein. Three independent cultures were used for both groups.

During infection, the expression pattern of PaTJ phage-encoding genes undergoes dynamic changes. To identify the early-expressed genes in the latent stage, transcriptome analysis was conducted on MPAO1 cells infected within the PaTJ phage (MOI = 0.01) for 30 min or not. In this stage, those genes involved in DNA replication (DNA primase and DNA polymerase), recombination (integrase), and repair (helicases) become highly expressed to enable the phage to hijack the host’s cellular machinery for its own replication (Figure 6). In addition, those genes encoding structural proteins (major capsid protein, tail fiber-related proteins, and other diverse structural phage proteins), packaging (terminase), and nucleases (endonuclease VII and exonuclease), are activated to facilitate the assembly of progeny phages and the eventual lysis and release of new viral particles. The majority of these genes are expressed in several clusters, suggesting the possibility that phage genes may share promoters to ensure coordinated regulation and efficient expression of functionally related genetic elements. In addition, 26 genes encoding lysin, holin, HicA toxin, endopeptidase, minor tail protein, diverse structural proteins, and several hypothetical proteins were not expressed at this stage and they may be genes expressed at late infection stages responsible for host lysis and the final assembly of phage particles inside the cells. This orchestrated pattern of gene expression ensures the efficient propagation of the phage within the host environment.

### 3.6. PaTJ Affects Host Metabolism During Infection

**Figure 7:**
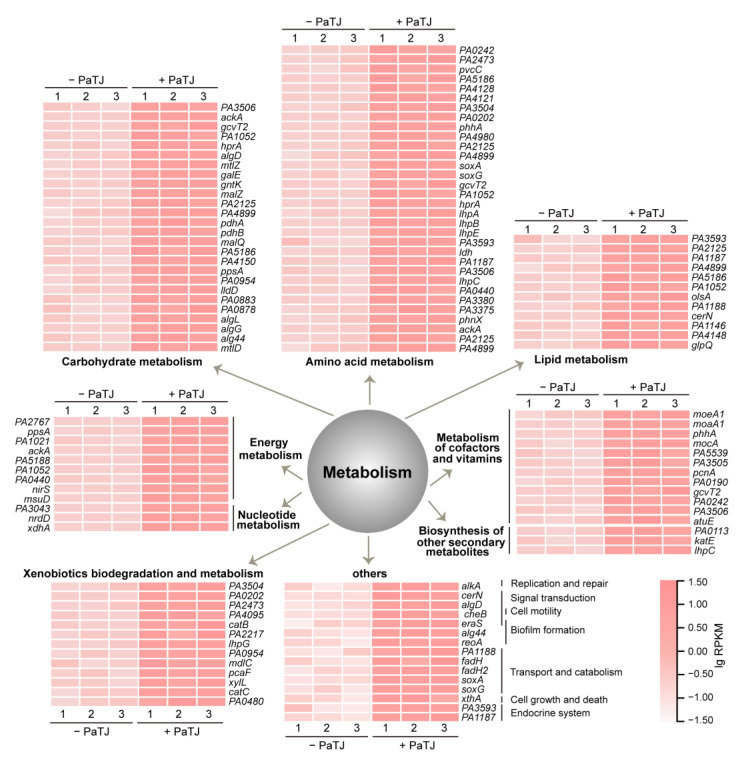
The impact of PaTJ infection on expression of host genes. Normalized expression of MPAO1 genes in samples in Figure 6 was also analyzed. Only KEGG pathways related to metabolism were significantly enriched and the expression levels (lgRPKM) of genes in enriched pathways were depicted in heatmaps. Three independent cultures were used for both groups.

During PaTJ infection, the expression of bacterial genes undergoes rapid and significant alterations as the host cell responds to the viral invasion. Surprisingly, neither the genes involved in stress responses as the bacterium recognizes the presence of foreign genetic material nor the genes related to defense mechanisms, such as restriction-modification systems and toxin-antitoxin systems, were significantly induced at the latent stage of PaTJ infection. However, genes involved in diverse KEGG metabolism pathways were significantly upregulated (Figure 7), with no pathway being downregulated during this process. This observation indicates that cells at the latent stage of phage infection are highly active. The upregulation of xenobiotics biodegradation and metabolism may represent a cellular response aimed at clearing phage DNA and its expression products. Additionally, in order to survive the threat of phages, cells accelerated the utilization of intracellular nutrients, as evidenced by the upregulation of carbohydrate metabolism, amino acid metabolism, lipid metabolism, nucleotide metabolism, energy metabolism, and metabolism of cofactors and vitamins, resulting in the upregulation of genes involved in the biosynthesis of other secondary metabolites. Furthermore, gene *alkA* associated with DNA replication and repair pathways also exhibited increased expression as the bacterium attempted to repair damage caused by the phage. Moreover, gene expression related to mechanisms involved in signal transduction, transport and catabolism, cell growth and death, and the endocrine system were also upregulated to limit phage propagation. This dynamic modulation of bacterial gene expression reflects the intricate interplay between the host and the infecting phage, ultimately shaping the outcome of the infection.

### 3.7. Potential of PaTJ in Biofilm Degradation

**Figure 8:**
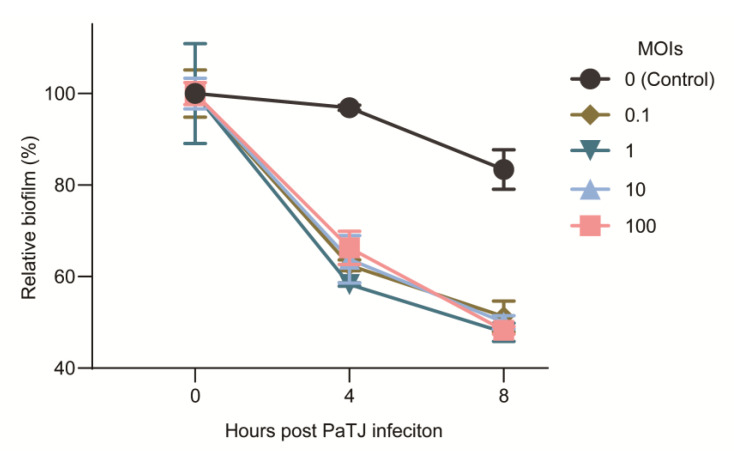
Biofilms in a static growth phase were exposed to PaTJ phages, and the residual biofilm was assessed at 0, 4, and 8 h post-infection. The relative biofilm was calculated by comparing it to the initial time point (time 0) when the PaTJ phages were introduced at different MOIs. Three independent cultures were used for each MOI and the data were presented as the mean ± standard deviation.

*P. aeruginosa* is known to form biofilms in vivo and in vitro, one of the most important virulence determinants and survival in a hypoxic atmosphere or other extremely harsh environments [26,37,38,39]. To assess the biofilm degradation potential of the PaTJ phage against pre-established biofilms, MPAO1 cells were allowed to form attached biofilm for 24 h, after which the PaTJ phage introduced a serial of initial MOIs of ranging from 0 to 100 to establish a phage–bacterial co-culture system. As shown in Figure 8, a significant loss of biomass was observed in all MOI groups except the control group without adding any phages, with approximately 50% biomass observed after 8 h of incubation even at a low MOI of 0.1. This suggests that PaTJ has a strong potential to the degradation of biofilm structure which may be related to its lysis of cells on the periphery of the biofilm, further penetrating bacterial biofilms to infect inner bacteria, resulting in in lysis.

## 4. Discussion

*P. aeruginosa* is currently listed as one of the top three critically resistant pathogens by the World Health Organization, and is predicted to cause 10 million deaths annually by 2050 [40]. The use of antibiotics in infection treatment has led to the emergence of multiple-drug-resistant *P. aeruginosa*, often resulting in chronic infections that are challenging to eradicate [2,3,4]. The emergency of antibiotic resistance is a consistent concerned, and all antibiotics used in clinical currently were detected to be resistant [3]. New antibiotics or therapeutic methods are in urgent need in clinics. Phage therapy stands out as one of the most promising strategies for addressing this global health threat [14]. In this study, the isolation and characterization of a potential lytic phage for *P. aeruginosa* phage therapy has provided significant insights into the modulation of gene expression and biofilm degradation.

The observation of the expression patterns of PaTJ encoding genes and the specific induction of host metabolism-related genes following phage infection underscores the potential impact of phage therapy on bacterial metabolic processes. These responses may not be universal across all hosts during viral infection. For instance, the infection by the temperate phage PaP3 (LUZ24-like virus) regulated the expression of 38% (2160/5633) genes, with amino acid metabolism being the most ‘vulnerable’ targets of these phage genes [41]. Additionally, within 15 min of infection by phage LUZ19, there was a sharp increase in 60% of all phage transcripts, but a complex pattern of bacterial up- and downregulated genes without protein degradation during phage infection, while also inducing the production of seven stress-related bacterial proteins [42]. Furthermore, infection by the single-stranded-RNA phage PRR1 resulted in changes in the expression levels of <4% of *P. aeruginosa* genes, which tend to be involved in one of three major cellular functions, i.e., transport, energy production, and protein synthesis [43]. Therefore, understanding the gene expression pattern of both phage and host genes during infection of each candidate phage in cocktails is essential, and the combination of diverse mechanisms may enhance the killing efficiency of the cocktail against the pathogen. While the isolation of the new phage PaTJ holds promise for inclusion in phage cocktails and future therapy applications, conducting evaluations of various phages using standardized protocols and the same *P. aeruginosa* host strain would enhance our comprehension of their lytic efficiency, gene expression profiles, and biofilm degradation capabilities.

*P. aeruginosa* forms biofilms that can coat mucosal surfaces or invasive devices, enabling bacterial habitats to escape antibiotics [44,45,46]. The demonstrated capability of the phage PaTJ in degrading *P. aeruginosa* biofilms is consistent with the growing body of research emphasizing the role of phages in disrupting biofilm, including phages DRL-P1 (*Pbunavirus*) [28], Phage_Pae01(*Pakpunavirus*) [24], PAcq-4 (*Pbunavirus*), and PAgz-2 (*Phikmvvirus*) [26]. These findings underscore the multifaceted impact of phage therapy on *P. aeruginosa* and contribute to a broader understanding of phage–bacteria interactions. It is well established that bacteria evolve as a response to antibiotics over eons. As observed in this study, continuous treatment of infected bacteria with *P. aeruginosa* phages can lead to the emergence of resistant mutant colonies, potentially resulting in a more challenging secondary infection to cure compared to the original infection. This observation could underscore the pressing need for the implementation of combinatorial phage therapy, which involves the simultaneous use of multiple phages to target bacterial infections, and prompt an in-depth exploration of diverse strategies to effectively combat and overcome resistance mechanisms that may arise. The PaTJ phage in this study utilized T4P as a receptor, and the emergence of mutant colonies may be attributed to the mutation of proteins comprising T4P, a phenomenon commonly observed during the selection of phage-resistant mutants during phage infection [47]. Therefore, the consideration of using phages with different receptors in the preparation of cocktails may enhance the efficiency of phage therapy. Furthermore, investigating the long-term evolutionary implications of phage therapy on bacterial populations, including the emergence of resistance and the stability of gene expression alterations, will be crucial for advancing the clinical translation of phage–based interventions. Over the past two decades, scientific research and hospital efforts have identified and characterized a series of lytic phages belonging to different families, demonstrating potential in phage therapy for *P. aeruginosa* infection [24,28,41,42,43]. However, these limited reported phages are not all deposited in a public library of phages as resources for cocktails. The isolation of more phages and the characterizing their morphology and genomic phenotypes are necessary, and the integration into a library is a long-term endeavor. Moreover, scientists have been genetically engineering phages using synthetic biology approaches to enhance their killing efficiency. Therefore, more fundamental and applied efforts are necessary before these therapies become accessible to the public on a larger scale.

The PaTJ phage in this study carries genes that may function in maintaining the lysogeny of prophages, including repressor and integrase, which may integrate it as a prophage in the host cell. The expression of prophage structural genes has been shown to be involved in phage defense [35] and prophages encoded accessory genes, including toxin-antitoxins systems, also exhibit phage defense activity [26,35,48,49,50,51,52]. Furthermore, PaTJ encodes both endonuclease and exonuclease, along with many other accessory genes with unknown functions, which may play a role in phage-host interaction. When preparing a cocktail, the effects of host-carried prophages and other mobile genetic elements and their encoding of antiphage systems should also be considered. Future research should aim to delve deeper into specific mechanisms underlying phage-induced gene expression changes and biofilm degradation. Ultimately, these research avenues hold the potential to further refine and expand the applications of phage therapy in addressing complex bacterial infections and biofilm-associated diseases.

In this study, we provided several characteristics of a newly isolated phage PaTJ infecting *P. aeruginosa*. While we identified the expression patterns of phage and host genes at the end of the latent stage during infection, we did not conduct direct associations between transcriptomic findings and specific infection stages due to the potent infection ability of PaTJ. This limitation hindered a more detailed understanding of how PaTJ influences host pathways over time. Furthermore, although we explored the potential applications and limitations of utilizing PaTJ, the study did not assess the effectiveness of PaTJ in phage therapy for clinical multidrug-resistant strains.

## 5. Conclusions

In this study, we isolated and characterized a lytic phage, PaTJ, targeting *P. aeruginosa,* which utilizes T4P as a receptor. PaTJ is classified within the phage family *Mesyanzhinovviridae*, displaying a relatively short latent period and a high burst size. Genomic analysis indicated the absence of antibiotic-resistant and virulence genes, highlighting its potential as a candidate for controlling the formation of *P. aeruginosa* biofilm. Transcriptome analysis revealed a dynamic expression pattern of phage-encoding genes during PaTJ infection, and it induced metabolism pathways specifically during the latent infection period. However, the presence of lysogeny-related repressor and integrase in PaTJ suggests the need for future studies to determine its lysogenic potential and in vivo therapy efficacy.

## Data Availability

The whole genome sequence of phage PaTJ was deposited in NCBI’s GenBank Database with accession number OM809160. The raw data for transcriptome results of PaTJ infection cells were deposited in the Sequence Read Archive (SRA) under BioProject accession number PRJNA1137540. All the other supporting data are available in the paper and Supplementary Materials.

## Supplementary Materials

The following supporting information can be downloaded at: https://www.mdpi.com/article/10.3390/v16121816/s1, Table S1: Bacterial strains, plasmids, and primers used in this study. R indicates resistance, Gm indicates gentamycin; Table S2: Annotation of PaTJ; Table S3: Information of *Pseudomonas* phages for the maximum likelihood tree.
