# Supplementary material for: Isolation and Characterization of a Lytic Phage PaTJ Against Pseudomonas aeruginosa
Source: Viruses. 2024 Nov 21;16(12):1816. doi: 10.3390/v16121816 (PMC11680426; doi:10.3390/v16121816)
Supplement: Supplementary file 1 [file viruses-16-01816-s001.zip › Table S1.pdf]

**Table S1. Bacterial strains, plasmids and primers used in this study.** Gm indicates gentamycin.

| | Description | Source |
|---|---|---|
| **Strains** | | |
| MPAO1 | wild-type | [26] |
| Δ*pilC* | *pilC* removed from MPAO1 host chromosome | this study |
| Δ*pilA* | *pilA* removed from MPAO1 host chromosome | this study |
| WM3064 | W. Metcalf, UIUC | |
| **Plasmids** | | |
| pEX18Gm | $Gm^R$, *oriT*$^+$, *sacB*$^+$, gene replacement vector | [29] |
| pEX18Gm-*pilC*-up-down | $Gm^R$, for deleting *pilC* | this study |
| pEX18Gm-*pilA*-up-down | $Gm^R$, for deleting *pilA* | this study |
| **Primers for gene knockout** | | |
| *pilC*-up-F | acgacggccagtgccaagcttTCTGCTCGTCTCAAGGTAAT | |
| *pilC*-up-R | ATGGCTGGCCAGGTAGTCGAGGAGGGGCATGGATTAATCCTTGGTCACGCGGTTGACTT | |
| *pilC*-down-F | AAGTCAACCGCGTGACCAAGGATTAATCCATGCCCCTCCTCGACTACCTGGCCAGCCAT | |
| *pilC*-down-R | tatgaccatgattacgaattcAGTTGGTGATCGGCATCGAT | |
| *pilC*-SF | GAGCAAGCCCGCAAAGAAG | |
| *pilC*-SR | CCAGTTGCGCTCCATCATCT | |
| *pilC*-LF | AAGATGCTGCTGGATGCCAT | |
| *pilC*-LR | GATCAGCGGGTGCAGCAATT | |
| *pilA*-up-F | ACGACGGCCAGTGCCAAGCTTTCGTTCGGAGATATCCAGGCC | |
| *pilA*-up-R | GATGGCTAAACAAGCCACCTTCGATCACCGAATCTCTCCGTTGATTATGTATAGGCCTA | |
| *pilA*-down-F | TAGGCCTATACATAATCAACGGAGAGATTCGGTGATCGAAGGTGGCTTGTTTAGCCATC | |
| *pilA*-down-R | TATGACCATGATTACGAATTCATGCCTAACCTCACCCTTGCC | |
| *pilA*-SF | AATGTCACATCCTGTCCGTTTT | |
| *pilA*-SR | AACCCCCGACCTTCTCATTA | |
| *pilA*-LF | TTGCAGTGCGAACAGAGTTTTC | |
| *pilA*-LR | GGTTGCCACAACCATCGCATCG | |

**References:**


26. Guo, Y.; Tang, K.; Sit, B.; Gu, J.; Chen, R.; Shao, X.; Lin, S.; Huang, Z.; Nie, Z.; Lin, J.; Liu, X.; Wang, W.; Gao, X.; Liu, T.; Liu, F.; Luo, H. R.; Waldor, M. K.; Wang, X., Control of lysogeny and antiphage defense by a prophage-encoded kinase-phosphatase module. *Nat. Commun.* **2024,** 15, (1).

29. Hoang, T. T.; Karkhoff-Schweizer, R. R.; Kutchma, A. J.; Schweizer, H. P., A broad-host-range Flp-*FRT* recombination system for site-specific excision of chromosomally-located DNA sequences: application for isolation of unmarked *Pseudomonas aeruginosa* mutants. *Gene* **1998,** 212, (1), 77-86.
